# *catena*-Poly[[[diaqua­(1,10-phenanthroline-κ^2^ *N*,*N*′)cobalt(II)]-μ-1*H*-benzimidazole-5,6-dicarboxyl­ato-κ^2^ *N* ^3^:*O* ^6^] sesquihydrate}

**DOI:** 10.1107/S1600536812043760

**Published:** 2012-11-03

**Authors:** Dong-Bo Xu, Yu Fang, De-Li Jiang, Yu Zhu, Min Chen

**Affiliations:** a School of Chemistry and Chemical Engineering, Jiangsu University, Zhenjiang 212013, People’s Republic of China

## Abstract

In the title compound, {[Co(C_9_H_4_N_2_O_4_)(C_12_H_8_N_2_)(H_2_O)_2_]·1.5H_2_O}_*n*_, the Co^II^ atom is hexa­coordinated by one N atom and one O atom from two symmetry-related 1*H*-benzimidazole-5,6-dicarboxyl­ate ligands, two N atoms from one 1,10-phenanthroline ligand (phen) and two water mol­ecules. The dihedral angle between the 1*H*-benzimidazole-5,6-dicarboxyl­ate and 1,10-phenanthroline ligands is 74.41 (4)°. The crystal packing is governed by inter­molecular O—H⋯O and N—H⋯O hydrogen-bonding inter­actions. All water (coordin­ating and lattice) mol­ecules take part in the hydrogen-bonding inter­actions. In addition, there are π–π stacking inter­actions between inversion-related phen ligands, the shortest centroid–centroid distance being 3.7536 (16) Å. One of the two lattice water molecules shows half-occupancy.

## Related literature

For general background to 1*H*-benzoimidazole-5,6-dicarboxyl­ate complexes, see: Lo *et al.* (2007 ▶); Gao *et al.* (2008 ▶); Yao *et al.* (2008 ▶). For 1,10-phenanthroline as a bridging ligand, see: Chesnut *et al.* (1999 ▶). For a similar structure with Ni^II^, see: Song *et al.* (2009 ▶).
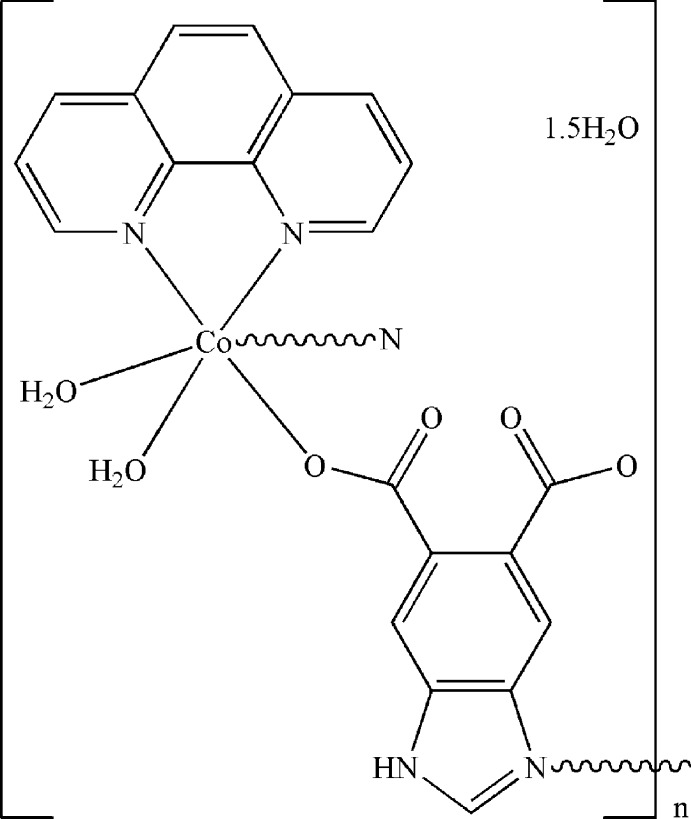


## Experimental

### 

#### Crystal data

[Co(C_9_H_4_N_2_O_4_)(C_12_H_8_N_2_)(H_2_O)_2_]·1.5H_2_O
*M* *_r_* = 506.33
Monoclinic, 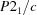

*a* = 9.7250 (11) Å
*b* = 11.3956 (13) Å
*c* = 19.296 (2) Å
β = 103.109 (2)°
*V* = 2082.7 (4) Å^3^
*Z* = 4
Mo *K*α radiation
μ = 0.88 mm^−1^
*T* = 173 K
0.30 × 0.24 × 0.20 mm

#### Data collection

Rigaku Saturn724+ diffractometer
Absorption correction: multi-scan (*CrystalClear*; Rigaku, 2008 ▶) *T*_min_ = 0.776, *T*_max_ = 0.838
17888 measured reflections
4797 independent reflections
3761 reflections with *I* > 2σ(*I*)
*R*_int_ = 0.043

#### Refinement

*R*[*F* ^2^ > 2σ(*F* ^2^)] = 0.036
*wR*(*F* ^2^) = 0.090
*S* = 1.05
4796 reflections
331 parameters
12 restraints
H-atom parameters constrained
Δρ_max_ = 0.42 e Å^−3^
Δρ_min_ = −0.49 e Å^−3^

### 

Data collection: *CrystalClear* (Rigaku, 2008 ▶); cell refinement: *CrystalClear*; data reduction: *CrystalClear*; program(s) used to solve structure: *SHELXS97* (Sheldrick, 2008 ▶); program(s) used to refine structure: *SHELXL97* (Sheldrick, 2008 ▶); molecular graphics: *SHELXTL* (Sheldrick, 2008 ▶); software used to prepare material for publication: *SHELXTL*.

## Supplementary Material

Crystal structure: contains datablock(s) global, I. DOI: 10.1107/S1600536812043760/zq2183sup1.cif

Structure factors: contains datablock(s) I. DOI: 10.1107/S1600536812043760/zq2183Isup2.hkl

Additional supplementary materials:  crystallographic information; 3D view; checkCIF report

## Figures and Tables

**Table 1:** Selected bond lengths (Å)

N2—Co1^i^	2.1304 (17)
N3—Co1	2.1412 (18)
N4—Co1	2.1478 (18)
O4—Co1	2.0582 (14)
O*W*1—Co1	2.1859 (15)
O*W*2—Co1	2.0689 (16)

Symmetry codes: (i) 
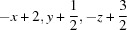
; (ii) 
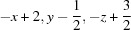
.

**Table 2:** Hydrogen-bond geometry (Å, °)

*D*—H⋯*A*	*D*—H	H⋯*A*	*D*⋯*A*	*D*—H⋯*A*
O*W*1—H1*C*⋯O3	0.85	1.83	2.650 (2)	160
O*W*2—H2*D*⋯O*W*3	0.83	1.88	2.693 (2)	164
N1—H1*A*⋯O*W*1^iii^	0.86	2.05	2.837 (2)	151
O*W*1—H1*D*⋯O2^iv^	0.85	1.82	2.654 (2)	168
O*W*2—H2*C*⋯O1^iv^	0.86	1.77	2.619 (2)	172 (3)
O*W*3—H3*C*⋯O3^v^	0.87	1.92	2.726 (3)	155 (3)
O*W*3—H3*D*⋯O*W*4^vi^	0.84	2.38	2.940 (5)	125
O*W*4—H4*C*⋯O*W*3^vii^	0.85	2.10	2.891 (4)	154 (5)
O*W*4—H4*C*⋯O*W*2^vii^	0.85	2.54	3.166 (4)	131 (5)
O*W*4—H4*D*⋯O1^ii^	0.86	2.06	2.837 (4)	151

Symmetry codes: (ii) 
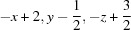
; (iii) 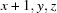; (iv) 
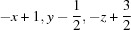
; (v) 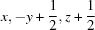; (vi) 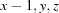; (vii) 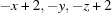.

**Fig. 1.**
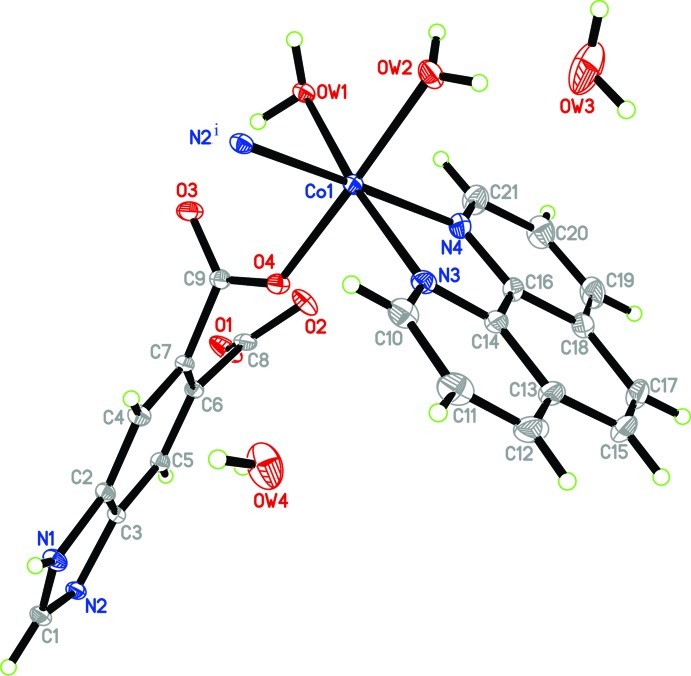
The molecular structure of the title compound with displacement ellipsoids drawn at the 30% probability level [H-atoms are shown as spheres of arbitrary size; symmetry code: (i) = x+2, y-0.5, -z+1.5].

**Fig. 2.**
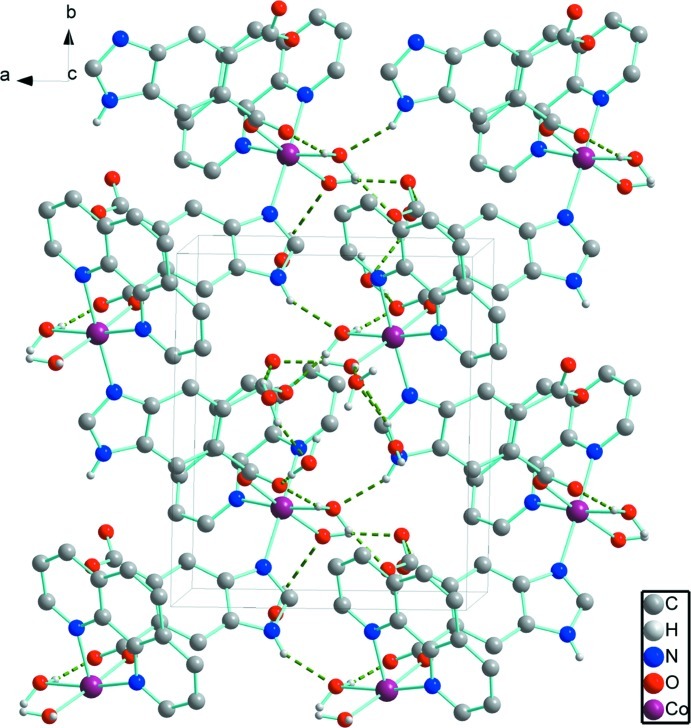
A view of the two-dimensional layer constructed by O-H···O and N-H···O hydrogen bonding interactions.

## supplementary crystallographic information

### Comment

The design and construction of supramolecular architectures have received considerable attention in recent years, in the structural investigation of 1*H*-benzimidazole-5,6-dicarboxylate complexes, it has been found that 1*H*-benzimidazole-5,6-dicarboxylate acid can function as a multidentate ligand (Lo *et al.*, 2007; Gao *et al.*, 2008; Yao *et al.*, 2008), with versatile binding and coordination modes. 1,10-phenanthroline is also a good example for a bridging ligand that can link metal centers into extended networks, and a number of one-, two- and three- dimensional metal-1,10-phenanthroline frameworks have been generated (Chesnut *et al.*, 1999). According to the procedure by Song *et al.* (Song *et al.*, 2009), the reaction of 1*H*-benzimidazole-5,6-dicarboxylate acid with cobalt chloride in an alkaline aqueous solution yielded the Co^II^ coordination polymer whose the crystal structure is reported here.

As illustrated in Fig. 1, the Co^II^ atom exhibits a slightly distorted octahedral coordination sphere, defined by one N atom and one O atom [Co1^i^—N2 = 2.1304 (17) Å, Co1—O4 = 2.0582 (14) Å] from two different 1*H*-benzimidazole-5,6-dicarboxylate ligands, two N atoms [Co1—N3 = 2.1412 (18) Å, Co1—N4 = 2.1478 (18) Å] from one 1,10-phenanthroline ligand and two water molecules [Co1—OW1 = 2.1859 (15) Å, Co1—OW2 = 2.0689 (16) Å]. The metal atoms are linked by bidentate 1*H*-benzimidazole-5,6-dicarboxylate groups into one dimensional chain. Inter/intramolecular O—H···O and N—H···O hydrogen bonds between the carboxylate O atoms of 1*H*-benzimidazole-5,6-dicarboxylate and the coordinated water and solvent water molecules lead to a two-dimensional layer (Fig. 2). The layers are further self-assembled into a three-dimensional supramolecular network by intermolecular N-H···O hydrogen bonds between the imidazole units and carboxylate groups (Table 1). In the crystal structure, π-π stacking interactions between inversion-related phen ligands are also observed with a shortest centroid-centroid distance of 3.7536 (16) Å [between (N4/C16/C18/C19/C20/C21) and (N4/C16/C18/C19/C20/C21)^viii^ with (viii) = 1-*x*, 1-*y*, 2-*z*)].

### Experimental

According to the procedure by Song *et al.* (2009), a mixture of cobalt chloride (0.2 mmol), 1*H*-benzimidazole-5,6-dicarboxylate acid (0.2 mmol), 1,10-phenanthroline (0.2 mmol), NaOH (0.1 mmol) and H_2_O (15 mL) was placed in a 25 mL Teflon reactor, which was heated to 413 K for four days and then cooled to room temperature at a rate of 10 K h^-1^. The crystals obtained were washed with water and dryed in air.

### Refinement

Carbon and nitrogen bound H atoms were placed at calculated positions and were treated as riding on the parent C or N atoms with C—H = 0.93 Å, N—H = 0.86 Å, and *U*_iso_(H) = 1.2*U*_eq_ (C,N). The water H-atoms were located in a difference Fourier map, and were refined with distance restraint of O—H = 0.85 (2) Å and *U*_iso_(H) = 1.5*U*_eq_ (O).

### Crystal data

**Table:**

[Co(C_9_H_4_N_2_O_4_)(C_12_H_8_N_2_)(H_2_O)_2_]·1.5H_2_O	*F*(000) = 1040
*M**_r_* = 506.33	*D*_x_ = 1.615 Mg m^−^^3^
Monoclinic, *P*2_1_/*c*	Mo *K*α radiation, λ = 0.71073 Å
Hall symbol: -P 2ybc	Cell parameters from 4850 reflections
*a* = 9.7250 (11) Å	θ = 2.1–27.5°
*b* = 11.3956 (13) Å	µ = 0.88 mm^−^^1^
*c* = 19.296 (2) Å	*T* = 173 K
β = 103.109 (2)°	Prism, red
*V* = 2082.7 (4) Å^3^	0.30 × 0.24 × 0.20 mm
*Z* = 4	

### Data collection

**Table:**

Rigaku Saturn724+ diffractometer	4797 independent reflections
Radiation source: fine-focus sealed tube	3761 reflections with *I* > 2σ(*I*)
Graphite monochromator	*R*_int_ = 0.043
ω scans	θ_max_ = 27.5°, θ_min_ = 2.1°
Absorption correction: multi-scan (*CrystalClear*; Rigaku, 2008)	*h* = −11→12
*T*_min_ = 0.776, *T*_max_ = 0.838	*k* = −14→14
17888 measured reflections	*l* = −25→25

### Refinement

**Table:**

Refinement on *F*^2^	Primary atom site location: structure-invariant direct methods
Least-squares matrix: full	Secondary atom site location: difference Fourier map
*R*[*F*^2^ > 2σ(*F*^2^)] = 0.036	Hydrogen site location: inferred from neighbouring sites
*wR*(*F*^2^) = 0.090	H-atom parameters constrained
*S* = 1.05	*w* = 1/[σ^2^(*F*_o_^2^) + (0.0355*P*)^2^ + 1.1164*P*] where *P* = (*F*_o_^2^ + 2*F*_c_^2^)/3
4796 reflections	(Δ/σ)_max_ = 0.002
331 parameters	Δρ_max_ = 0.42 e Å^−^^3^
12 restraints	Δρ_min_ = −0.49 e Å^−^^3^

### Special details

**Table:**

Geometry. All esds (except the esd in the dihedral angle between two l.s. planes) are estimated using the full covariance matrix. The cell esds are taken into account individually in the estimation of esds in distances, angles and torsion angles; correlations between esds in cell parameters are only used when they are defined by crystal symmetry. An approximate (isotropic) treatment of cell esds is used for estimating esds involving l.s. planes.
Refinement. Refinement of F^2^ against ALL reflections. The weighted R-factor wR and goodness of fit S are based on F^2^, conventional R-factors R are based on F, with F set to zero for negative F^2^. The threshold expression of F^2^ > 2sigma(F^2^) is used only for calculating R-factors(gt) etc. and is not relevant to the choice of reflections for refinement. R-factors based on F^2^ are statistically about twice as large as those based on F, and R- factors based on ALL data will be even larger.

### Fractional atomic coordinates and isotropic or equivalent isotropic displacement parameters (Å^2^)

**Table:**

	*x*	*y*	*z*	*U*_iso_*/*U*_eq_	Occ. (<1)
C1	1.3426 (2)	0.48546 (18)	0.68601 (11)	0.0196 (4)	
H1	1.4358	0.4878	0.6816	0.023*	
C2	1.1479 (2)	0.42156 (18)	0.71347 (10)	0.0162 (4)	
C3	1.1260 (2)	0.53227 (18)	0.68112 (10)	0.0158 (4)	
C4	1.0404 (2)	0.36045 (18)	0.73494 (11)	0.0184 (4)	
H4	1.0562	0.2867	0.7559	0.022*	
C5	0.9938 (2)	0.58516 (18)	0.66973 (10)	0.0166 (4)	
H5	0.9779	0.6581	0.6478	0.020*	
C6	0.8856 (2)	0.52623 (18)	0.69192 (10)	0.0155 (4)	
C7	0.9086 (2)	0.41394 (18)	0.72378 (10)	0.0162 (4)	
C8	0.7474 (2)	0.59071 (19)	0.68525 (11)	0.0202 (4)	
C9	0.7931 (2)	0.34525 (17)	0.74648 (11)	0.0173 (4)	
C10	0.9568 (2)	0.1841 (2)	0.99354 (12)	0.0285 (5)	
H10	0.9609	0.1120	0.9715	0.034*	
C11	1.0612 (3)	0.2105 (3)	1.05439 (13)	0.0372 (6)	
H11	1.1333	0.1573	1.0717	0.045*	
C12	1.0563 (3)	0.3156 (3)	1.08825 (13)	0.0393 (7)	
H12	1.1254	0.3346	1.1285	0.047*	
C13	0.9455 (3)	0.3945 (2)	1.06151 (12)	0.0327 (6)	
C14	0.8459 (2)	0.3620 (2)	0.99951 (11)	0.0243 (5)	
C15	0.9306 (3)	0.5065 (3)	1.09338 (13)	0.0416 (7)	
H15	0.9968	0.5290	1.1340	0.050*	
C16	0.7329 (2)	0.4407 (2)	0.96903 (11)	0.0242 (5)	
C17	0.8237 (3)	0.5794 (3)	1.06611 (14)	0.0420 (7)	
H17	0.8160	0.6504	1.0887	0.050*	
C18	0.7208 (3)	0.5495 (2)	1.00241 (13)	0.0310 (6)	
C19	0.6081 (3)	0.6230 (2)	0.96966 (15)	0.0392 (6)	
H19	0.5964	0.6958	0.9894	0.047*	
C20	0.5165 (3)	0.5876 (2)	0.90918 (15)	0.0380 (6)	
H20	0.4416	0.6354	0.8875	0.046*	
C21	0.5363 (3)	0.4778 (2)	0.87982 (13)	0.0321 (6)	
H21	0.4731	0.4545	0.8384	0.039*	
N1	1.28761 (18)	0.39420 (15)	0.71539 (9)	0.0195 (4)	
H1A	1.3311	0.3310	0.7321	0.023*	
N2	1.25154 (17)	0.57083 (15)	0.66427 (9)	0.0172 (4)	
N3	0.85195 (19)	0.25700 (17)	0.96580 (9)	0.0220 (4)	
N4	0.64096 (19)	0.40575 (16)	0.90847 (9)	0.0237 (4)	
O1	0.71519 (16)	0.66394 (16)	0.63497 (9)	0.0359 (4)	
O2	0.67695 (16)	0.57174 (14)	0.73083 (8)	0.0279 (4)	
O3	0.68891 (16)	0.31022 (14)	0.69976 (8)	0.0250 (4)	
O4	0.81500 (15)	0.32296 (12)	0.81218 (7)	0.0191 (3)	
OW1	0.51234 (15)	0.24220 (13)	0.77827 (8)	0.0184 (3)	
H1C	0.5615	0.2496	0.7472	0.028*	
H1D	0.4589	0.1824	0.7723	0.028*	
OW2	0.55560 (16)	0.15584 (16)	0.92303 (8)	0.0280 (4)	
H2C	0.4677	0.1657	0.9039	0.042*	
H2D	0.5734	0.1556	0.9673	0.042*	
OW3	0.5829 (3)	0.1182 (2)	1.06327 (10)	0.0635 (7)	
H3C	0.6403	0.1383	1.1027	0.095*	
H3D	0.5210	0.0701	1.0688	0.095*	
OW4	1.2843 (5)	0.0672 (3)	1.0004 (2)	0.0451 (10)	0.50
H4C	1.3167	0.0004	0.9924	0.068*	0.50
H4D	1.2609	0.1116	0.9637	0.068*	0.50
Co1	0.69156 (3)	0.23795 (2)	0.870080 (14)	0.01613 (9)	

### Atomic displacement parameters (Å^2^)

**Table:**

	*U*^11^	*U*^22^	*U*^33^	*U*^12^	*U*^13^	*U*^23^
C1	0.0132 (10)	0.0213 (11)	0.0250 (11)	0.0005 (8)	0.0060 (8)	−0.0015 (9)
C2	0.0125 (10)	0.0178 (11)	0.0182 (10)	0.0033 (8)	0.0030 (8)	−0.0001 (8)
C3	0.0138 (10)	0.0170 (10)	0.0175 (10)	−0.0015 (8)	0.0052 (8)	−0.0020 (8)
C4	0.0180 (10)	0.0161 (10)	0.0210 (10)	0.0003 (8)	0.0046 (8)	0.0028 (8)
C5	0.0161 (10)	0.0153 (10)	0.0185 (10)	0.0020 (8)	0.0039 (8)	0.0007 (8)
C6	0.0119 (10)	0.0168 (10)	0.0174 (10)	0.0010 (8)	0.0024 (8)	−0.0010 (8)
C7	0.0142 (10)	0.0184 (10)	0.0171 (10)	−0.0038 (8)	0.0057 (8)	−0.0022 (8)
C8	0.0133 (10)	0.0188 (11)	0.0284 (11)	−0.0023 (9)	0.0044 (9)	−0.0007 (9)
C9	0.0152 (10)	0.0137 (10)	0.0234 (10)	0.0012 (8)	0.0056 (8)	−0.0010 (8)
C10	0.0252 (12)	0.0360 (14)	0.0239 (11)	0.0017 (10)	0.0049 (10)	0.0073 (10)
C11	0.0267 (13)	0.0548 (18)	0.0270 (13)	0.0008 (12)	0.0000 (10)	0.0131 (12)
C12	0.0298 (14)	0.0624 (19)	0.0221 (12)	−0.0170 (13)	−0.0018 (10)	0.0036 (12)
C13	0.0300 (13)	0.0483 (16)	0.0196 (11)	−0.0168 (12)	0.0056 (10)	−0.0046 (11)
C14	0.0245 (12)	0.0306 (13)	0.0188 (11)	−0.0090 (10)	0.0070 (9)	−0.0028 (9)
C15	0.0444 (17)	0.0558 (18)	0.0243 (12)	−0.0224 (15)	0.0071 (12)	−0.0168 (12)
C16	0.0269 (12)	0.0262 (12)	0.0219 (11)	−0.0056 (10)	0.0107 (9)	−0.0048 (9)
C17	0.0540 (18)	0.0423 (17)	0.0350 (14)	−0.0205 (14)	0.0213 (13)	−0.0217 (12)
C18	0.0363 (14)	0.0304 (13)	0.0315 (13)	−0.0106 (11)	0.0185 (11)	−0.0100 (10)
C19	0.0492 (17)	0.0260 (13)	0.0501 (16)	−0.0004 (12)	0.0273 (14)	−0.0116 (12)
C20	0.0418 (15)	0.0285 (14)	0.0464 (16)	0.0112 (12)	0.0157 (13)	−0.0016 (12)
C21	0.0322 (14)	0.0299 (14)	0.0337 (13)	0.0068 (11)	0.0062 (11)	−0.0038 (11)
N1	0.0151 (9)	0.0175 (9)	0.0266 (9)	0.0037 (7)	0.0058 (7)	0.0038 (7)
N2	0.0122 (8)	0.0169 (9)	0.0230 (9)	−0.0003 (7)	0.0052 (7)	−0.0012 (7)
N3	0.0179 (9)	0.0298 (11)	0.0179 (9)	−0.0026 (8)	0.0030 (7)	0.0025 (8)
N4	0.0235 (10)	0.0254 (10)	0.0234 (9)	0.0014 (8)	0.0080 (8)	−0.0030 (8)
O1	0.0180 (8)	0.0454 (11)	0.0462 (11)	0.0108 (8)	0.0115 (8)	0.0256 (9)
O2	0.0198 (8)	0.0337 (9)	0.0343 (9)	0.0079 (7)	0.0149 (7)	0.0098 (7)
O3	0.0210 (8)	0.0329 (9)	0.0204 (8)	−0.0104 (7)	0.0030 (6)	0.0012 (7)
O4	0.0172 (7)	0.0199 (8)	0.0198 (7)	−0.0028 (6)	0.0036 (6)	0.0014 (6)
OW1	0.0139 (7)	0.0203 (8)	0.0216 (7)	−0.0007 (6)	0.0055 (6)	0.0004 (6)
OW2	0.0193 (8)	0.0442 (11)	0.0215 (8)	0.0020 (8)	0.0066 (7)	0.0014 (8)
OW3	0.101 (2)	0.0582 (16)	0.0250 (10)	0.0048 (13)	0.0016 (11)	−0.0050 (10)
OW4	0.057 (3)	0.031 (2)	0.053 (2)	0.0131 (19)	0.027 (2)	0.0075 (18)
Co1	0.01335 (15)	0.01823 (16)	0.01708 (14)	0.00037 (11)	0.00402 (10)	−0.00106 (11)

### Geometric parameters (Å, º)

**Table:**

C1—N2	1.318 (3)	C15—C17	1.342 (4)
C1—N1	1.352 (3)	C15—H15	0.9300
C1—H1	0.9300	C16—N4	1.360 (3)
C2—N1	1.387 (2)	C16—C18	1.414 (3)
C2—C4	1.395 (3)	C17—C18	1.439 (4)
C2—C3	1.402 (3)	C17—H17	0.9300
C3—C5	1.392 (3)	C18—C19	1.411 (4)
C3—N2	1.403 (2)	C19—C20	1.359 (4)
C4—C7	1.391 (3)	C19—H19	0.9300
C4—H4	0.9300	C20—C21	1.405 (3)
C5—C6	1.395 (3)	C20—H20	0.9300
C5—H5	0.9300	C21—N4	1.327 (3)
C6—C7	1.415 (3)	C21—H21	0.9300
C6—C8	1.512 (3)	N1—H1A	0.8600
C7—C9	1.513 (3)	N2—Co1^i^	2.1304 (17)
C8—O2	1.250 (3)	N3—Co1	2.1412 (18)
C8—O1	1.264 (3)	N4—Co1	2.1478 (18)
C9—O3	1.259 (2)	O4—Co1	2.0582 (14)
C9—O4	1.263 (2)	OW1—Co1	2.1859 (15)
C10—N3	1.330 (3)	OW1—H1C	0.85
C10—C11	1.400 (3)	OW1—H1D	0.85
C10—H10	0.9300	OW2—Co1	2.0689 (16)
C11—C12	1.370 (4)	OW2—H2C	0.86
C11—H11	0.9300	OW2—H2D	0.83
C12—C13	1.409 (4)	OW3—H3C	0.87
C12—H12	0.9300	OW3—H3D	0.84
C13—C14	1.408 (3)	OW4—H4C	0.85
C13—C15	1.438 (4)	OW4—H4D	0.86
C14—N3	1.369 (3)	Co1—N2^ii^	2.1304 (17)
C14—C16	1.437 (3)		
			
N2—C1—N1	113.57 (18)	C18—C17—H17	119.5
N2—C1—H1	123.2	C19—C18—C16	116.9 (2)
N1—C1—H1	123.2	C19—C18—C17	124.1 (2)
N1—C2—C4	132.52 (19)	C16—C18—C17	118.9 (2)
N1—C2—C3	105.23 (17)	C20—C19—C18	120.0 (2)
C4—C2—C3	122.24 (18)	C20—C19—H19	120.0
C5—C3—C2	120.06 (18)	C18—C19—H19	120.0
C5—C3—N2	130.47 (19)	C19—C20—C21	119.2 (2)
C2—C3—N2	109.46 (17)	C19—C20—H20	120.4
C7—C4—C2	117.49 (19)	C21—C20—H20	120.4
C7—C4—H4	121.3	N4—C21—C20	123.0 (2)
C2—C4—H4	121.3	N4—C21—H21	118.5
C3—C5—C6	118.51 (19)	C20—C21—H21	118.5
C3—C5—H5	120.7	C1—N1—C2	107.13 (17)
C6—C5—H5	120.7	C1—N1—H1A	126.4
C5—C6—C7	120.88 (18)	C2—N1—H1A	126.4
C5—C6—C8	117.26 (18)	C1—N2—C3	104.61 (17)
C7—C6—C8	121.72 (17)	C1—N2—Co1^i^	123.74 (14)
C4—C7—C6	120.81 (18)	C3—N2—Co1^i^	130.77 (13)
C4—C7—C9	116.55 (18)	C10—N3—C14	117.8 (2)
C6—C7—C9	122.64 (18)	C10—N3—Co1	128.73 (16)
O2—C8—O1	125.1 (2)	C14—N3—Co1	113.40 (14)
O2—C8—C6	118.34 (19)	C21—N4—C16	118.0 (2)
O1—C8—C6	116.54 (18)	C21—N4—Co1	128.41 (16)
O3—C9—O4	125.49 (19)	C16—N4—Co1	113.55 (15)
O3—C9—C7	119.11 (18)	C9—O4—Co1	130.78 (13)
O4—C9—C7	115.30 (18)	Co1—OW1—H1C	96
N3—C10—C11	123.2 (2)	Co1—OW1—H1D	116.4
N3—C10—H10	118.4	H1C—OW1—H1D	114.1
C11—C10—H10	118.4	Co1—OW2—H2C	114
C12—C11—C10	119.4 (2)	Co1—OW2—H2D	120.0
C12—C11—H11	120.3	H2C—OW2—H2D	113.4
C10—C11—H11	120.3	H3C—OW3—H3D	113.6
C11—C12—C13	119.3 (2)	H4C—OW4—H4D	114.7
C11—C12—H12	120.3	O4—Co1—OW2	176.10 (6)
C13—C12—H12	120.3	O4—Co1—N2^ii^	91.56 (6)
C14—C13—C12	117.7 (2)	OW2—Co1—N2^ii^	89.40 (7)
C14—C13—C15	118.7 (2)	O4—Co1—N3	91.15 (6)
C12—C13—C15	123.6 (2)	OW2—Co1—N3	92.41 (7)
N3—C14—C13	122.6 (2)	N2^ii^—Co1—N3	99.75 (7)
N3—C14—C16	117.51 (19)	O4—Co1—N4	88.60 (6)
C13—C14—C16	119.9 (2)	OW2—Co1—N4	90.59 (7)
C17—C15—C13	121.7 (2)	N2^ii^—Co1—N4	177.70 (7)
C17—C15—H15	119.1	N3—Co1—N4	77.95 (7)
C13—C15—H15	119.1	O4—Co1—OW1	90.32 (6)
N4—C16—C18	122.8 (2)	OW2—Co1—OW1	85.92 (6)
N4—C16—C14	117.5 (2)	N2^ii^—Co1—OW1	89.04 (6)
C18—C16—C14	119.7 (2)	N3—Co1—OW1	171.05 (6)
C15—C17—C18	121.0 (2)	N4—Co1—OW1	93.26 (6)
C15—C17—H17	119.5		

Symmetry codes: (i) −*x*+2, *y*+1/2, −*z*+3/2; (ii) −*x*+2, *y*−1/2, −*z*+3/2.

### Hydrogen-bond geometry (Å, º)

**Table:**

*D*—H···*A*	*D*—H	H···*A*	*D*···*A*	*D*—H···*A*
O*W*1—H1*C*···O3	0.85	1.83	2.650 (2)	160
O*W*2—H2*D*···O*W*3	0.83	1.88	2.693 (2)	164
N1—H1*A*···O*W*1^iii^	0.86	2.05	2.837 (2)	151
O*W*1—H1*D*···O2^iv^	0.85	1.82	2.654 (2)	168
O*W*2—H2*C*···O1^iv^	0.86	1.77	2.619 (2)	172 (3)
O*W*3—H3*C*···O3^v^	0.87	1.92	2.726 (3)	155 (3)
O*W*3—H3*D*···O*W*4^vi^	0.84	2.38	2.940 (5)	125
O*W*4—H4*C*···O*W*3^vii^	0.85	2.10	2.891 (4)	154 (5)
O*W*4—H4*C*···O*W*2^vii^	0.85	2.54	3.166 (4)	131 (5)
O*W*4—H4*D*···O1^ii^	0.86	2.06	2.837 (4)	151

Symmetry codes: (ii) −*x*+2, *y*−1/2, −*z*+3/2; (iii) *x*+1, *y*, *z*; (iv) −*x*+1, *y*−1/2, −*z*+3/2; (v) *x*, −*y*+1/2, *z*+1/2; (vi) *x*−1, *y*, *z*; (vii) −*x*+2, −*y*, −*z*+2.

## References

[bb1] Chesnut, D. J., Haushalter, R. C. & Zubieta, J. (1999). *Inorg. Chim. Acta*, **292**, 41–51.

[bb2] Gao, Q., Gao, W.-H., Zhang, C.-Y. & Xie, Y.-B. (2008). *Acta Cryst.* E**64**, m928. 10.1107/S1600536808017595 PMC2961800 21202783

[bb3] Lo, Y.-L., Wang, W.-C., Lee, G.-A. & Liu, Y.-H. (2007). *Acta Cryst.* E**63**, m2657–m2658.

[bb4] Rigaku (2008). *CrystalClear* Rigaku Corporation, Tokyo, Japan.

[bb5] Sheldrick, G. M. (2008). *Acta Cryst.* A**64**, 112–122. 10.1107/S0108767307043930 18156677

[bb6] Song, W.-D., Wang, H., Hu, S.-W., Qin, P.-W. & Li, S.-J. (2009). *Acta Cryst.* E**65**, m701. 10.1107/S1600536809019680 PMC2969534 21583055

[bb7] Yao, Y. L., Che, Y. X. & Zheng, J. M. (2008). *Cryst. Growth Des.* **8**, 2299–2306.

